# Unexploded Ordnance in Civilian Settings: A Hidden Cause of Blast‐Related Trauma

**DOI:** 10.1002/ccr3.72051

**Published:** 2026-02-14

**Authors:** Chukwuka Elendu, Dependable C. Amaechi, Tochi C. Elendu, Emmanuel C. Amaechi, Ijeoma D. Elendu

**Affiliations:** ^1^ Federal University Teaching Hospital Owerri Nigeria; ^2^ Igbinedion University Okada Nigeria; ^3^ Imo State University Owerri Nigeria; ^4^ Madonna University Elele Nigeria; ^5^ Gregory University Uturu Nigeria

**Keywords:** blast injury prevention, civilian safety, hazard recognition, public health risk, unexploded ordnance

## Abstract

Clinicians should maintain a high index of suspicion when unfamiliar metallic objects are encountered in civilian settings. Early recognition of potential explosive hazards, avoidance of civilian handling, and prompt notification of appropriate authorities are essential to prevent secondary injury and reduce avoidable morbidity at both individual and community levels.

## 1 Case Description

During routine civilian activities in a semi‐rural community, multiple unexploded ordnance (UXO) devices were incidentally identified in non‐military settings over a six‐month period and brought to clinical attention through community reports to local healthcare personnel, who recognized the potential for blast‐related injury and initiated appropriate risk reporting.

**FIGURE 1:**
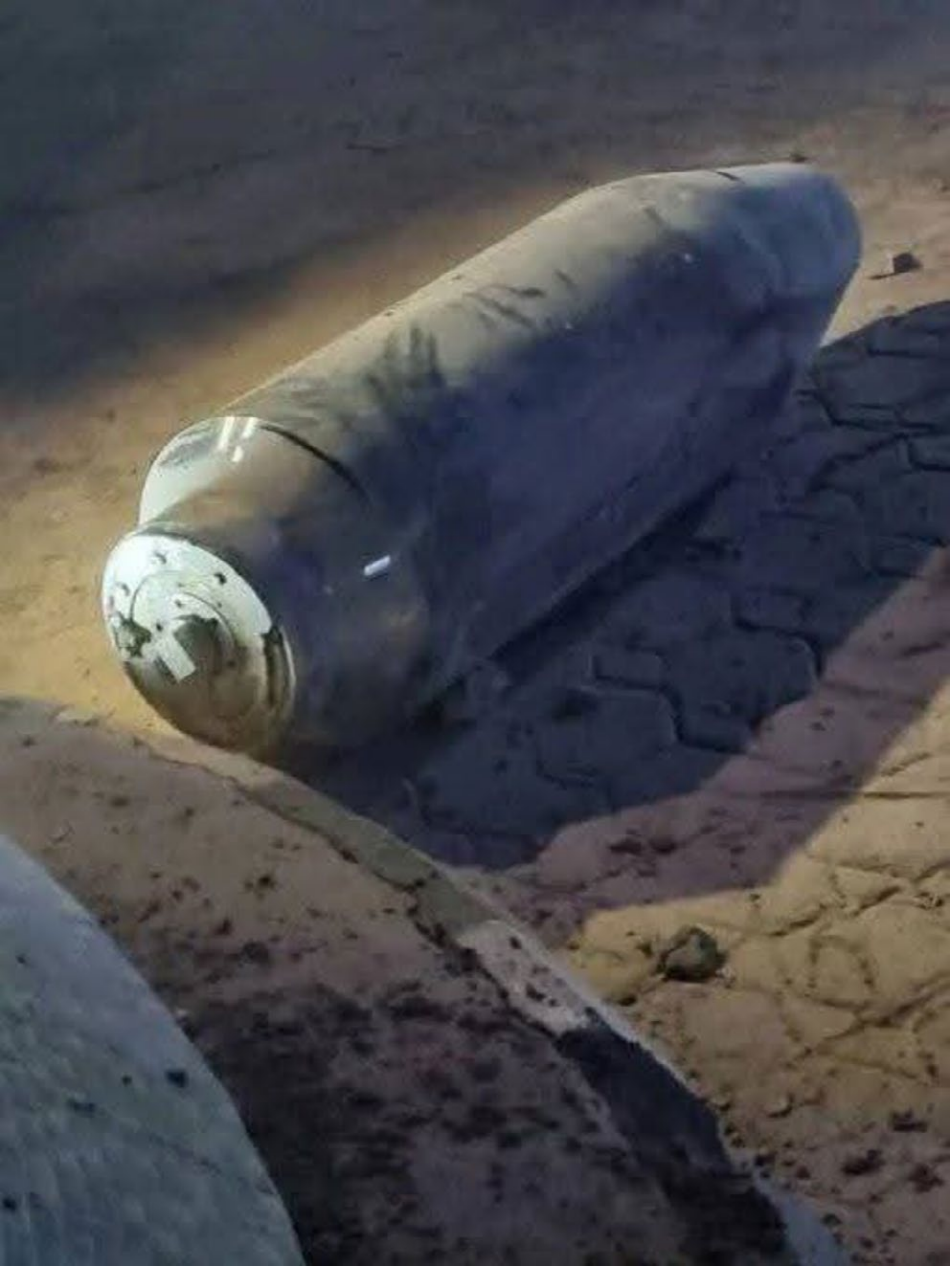
UXO lying openly on paved interlocking stones within a residential compound, with an intact cylindrical metallic casing and visible surface wear, illustrating the unexpected presence of a potential explosive hazard in a civilian setting.

The initial discovery occurred within the compound of a residential property, where occupants observed a large, metallic, cylindrical object lying openly on paved interlocking stones, which had been present for a prolonged period and was initially assumed to be inert scrap material. The device appeared intact, with visible structural components and surface wear, and showed no evidence of burial or concealment (Figure 1).

**FIGURE 2:**
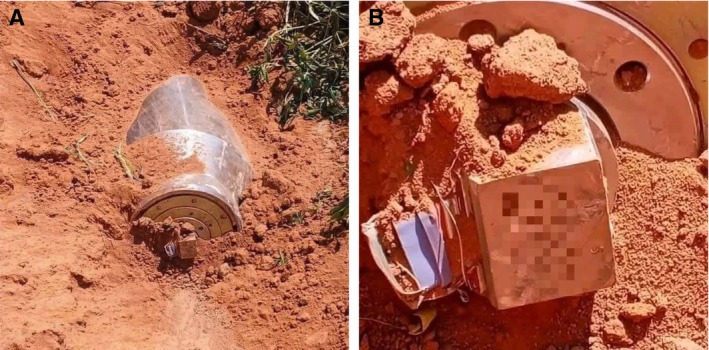
(A) UXO is partially embedded in loose lateritic soil, with only the posterior section exposed following shallow ground disturbance, highlighting the risk of inadvertent civilian contact during routine land activities. (B) Close‐up view of the exposed base and surface markings of the partially buried UXO, demonstrating identifiable manufactured components consistent with an engineered explosive device.

Subsequently, during shallow ground disturbance, another item of ordnance was encountered partially embedded in loose lateritic soil. Only the posterior section was visible at the time of discovery, with partial exposure likely related to soil erosion and recent surface disturbance. Following clinician‐led recognition of potential risk, activities were immediately suspended and access to the surrounding area restricted (Figure 2A), with close‐up visualization of the base and surface markings shown in Figure 2B.

**FIGURE 3:**
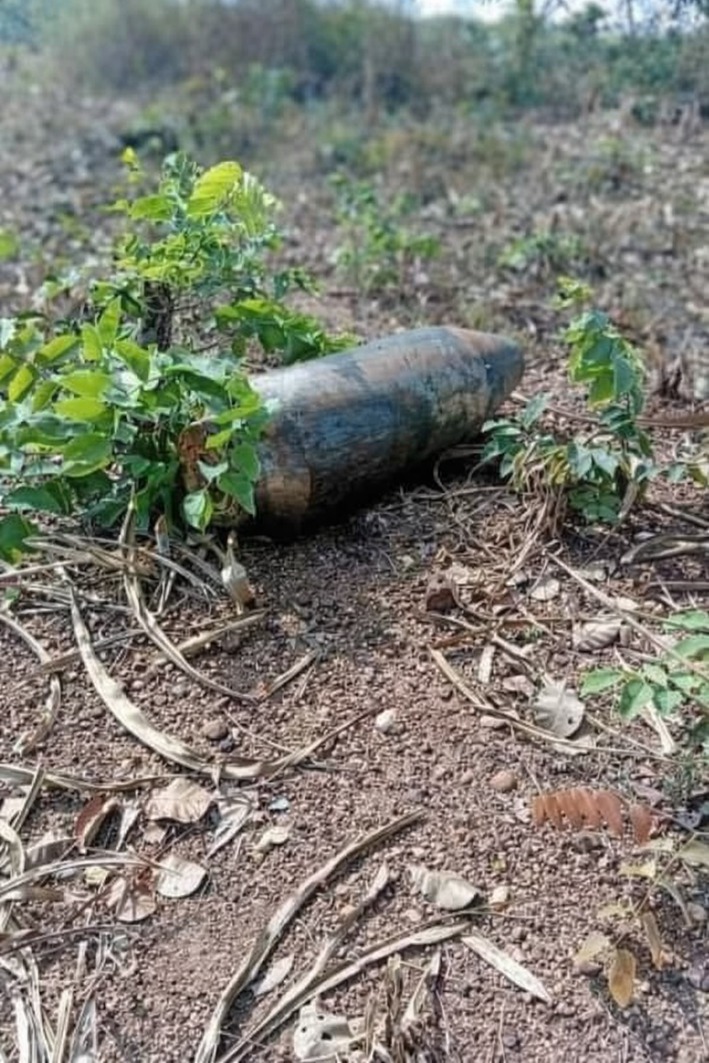
UXO resting on the ground surface among low vegetation on cultivated farmland, with much of the external casing exposed, illustrating civilian exposure to explosive hazards during routine agricultural activities.

**FIGURE 4:**
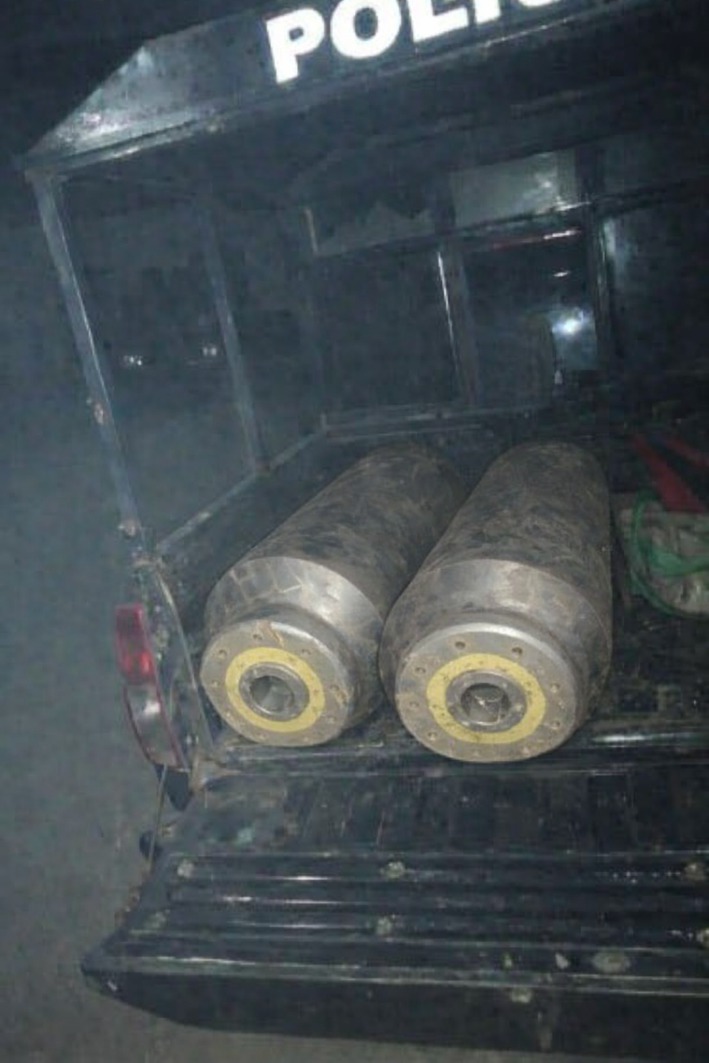
UXO devices following assessment by security and explosive ordnance disposal personnel, shown secured in a designated vehicle prior to controlled removal from the civilian environment.

Additionally, a separate device of ordnance was identified on cultivated farmland during routine agricultural work. It was lying horizontally among low vegetation, with much of its external casing exposed, consistent with surface exposure in an agricultural setting. Local farmers reported long‐term awareness of the object but had not previously associated it with explosive risk (Figure 3). Upon notification, security and explosive ordnance disposal personnel assessed and confirmed it as UXO and safely secured and removed it (Figure 4), with no detonation or injuries before formal intervention.

## 2 Discussion

The clinical concern raised by these images is that apparently inert objects encountered in residential or agricultural environments may represent active UXO, posing a significant risk of severe blast‐related injury if disturbed. UXO may persist in civilian settings for decades due to legacy munitions from historical armed conflict, abandoned military training activities, or improper disposal of explosive materials, even in areas not currently recognized as conflict zones. Such residual hazards are increasingly reported in low‐resource and post‐conflict regions, where land reuse and population growth expand civilian exposure [1].

Epidemiological data indicate that civilian UXO‐related incidents account for substantial morbidity and mortality worldwide, with children and agricultural workers disproportionately affected. Injuries are often severe, involving complex polytrauma resulting from combined primary, secondary, tertiary, and quaternary blast mechanisms, frequently exceeding the severity of conventional trauma [2]. These injury patterns underscore the importance of early clinical recognition and anticipatory management, even before detonation occurs.

From a clinical perspective, healthcare providers are essential in recognizing potential explosive hazards, initiating scene safety measures, and activating appropriate reporting and escalation pathways. As summarized in Table 1, key actions and “do's and don'ts” for healthcare workers are outlined to prevent avoidable injury. Together, these images highlight the need for sustained clinical vigilance and public health strategies informed by global UXO risk patterns to mitigate UXO‐related harm in civilian environments [2, 3].

**TABLE 1:** Suggested clinical action steps for healthcare workers when suspected UXO is reported in civilian settings.

Step	Clinical action	Key considerations for healthcare workers
1. Initial recognition	Identify reported object as potentially hazardous	Treat unfamiliar metallic, cylindrical, or military‐appearing objects as UXO until proven otherwise
2. Scene safety	Advise immediate cessation of activity and evacuation of the immediate area	Maintain a safe distance; prevent crowding or curiosity‐driven handling
3. Do not manipulate	Avoid physical handling, movement, or examination of the object	Do not touch, transport, disassemble, or attempt to neutralize the device
4. Avoid clinical procedures	Do not perform imaging, testing, or sampling on the object	Radiography, CT, or other imaging may pose detonation risk and should not be attempted
5. Notification and escalation	Initiate prompt notification of appropriate authorities	Contact security services and explosive ordnance disposal units through established reporting pathways
6. Risk communication	Provide clear guidance to civilians and bystanders	Emphasize danger, restrict access, and discourage further disturbance
7. Documentation	Record the incident and actions taken	Document time, location, reporting pathway, and referrals for public health follow‐up

## Author Contributions

**Chukwuka Elendu:** conceptualization, investigation, project administration, supervision, validation, visualization, writing – original draft. **Dependable C. Amaechi:** data curation, methodology, writing – review and editing. **Tochi C. Elendu:** data curation, writing – review and editing. **Emmanuel C. Amaechi:** funding acquisition, validation, writing – review and editing. **Ijeoma D. Elendu:** visualization, writing – review and editing.

## Funding

The authors have nothing to report.

## Disclosure

The views expressed in this report are solely those of the author(s) and do not represent the official positions of any affiliated institutions.

## Ethics Statement

The authors have nothing to report.

## Consent

Written informed consent was obtained for publication of this report and any accompanying images. No patient‐identifiable information or personal clinical data are included. Where applicable, permission was obtained from property owners for environmental images.

## Conflicts of Interest

The authors declare no conflicts of interest.

## Data Availability

The authors have nothing to report.
